# A method for noninvasive beat‐by‐beat visualization of His bundle signals

**DOI:** 10.1111/anec.13076

**Published:** 2023-07-26

**Authors:** S. Sengottuvel, S. Shenbaga Devi, M. Sasikala, S. Satheesh, R. J. Selvaraj

**Affiliations:** ^1^ SQUIDs Applications Section, SQUID and Detector Technology Division Materials Science Group, Indira Gandhi Centre for Atomic Research Kalpakkam India; ^2^ Department of Electronics and Communication Engineering, Centre for Medical Electronics Anna University Chennai India; ^3^ Department of Cardiology Jawaharlal Institute of Postgraduate Medical Education and Research Puducherry India

**Keywords:** His bundle signals, HV interval, interval‐dependent wavelet thresholding, magnetocardiography, signal space projection

## Abstract

**Background:**

Invasive recording of His bundle signals (HBS) in electrophysiological study (EPS) is important in determining HV interval, the time taken to activate the ventricles from the His bundle. Noninvasive surface measurements of HBS are attempted by averaging typically 100–200 cardiac cycles of ECG time series in body surface potential mapping (BSPM) and in magnetocardiography (MCG) which records weak cardiac magnetic fields by highly sensitive detectors. However, noninvasive beat‐by‐beat extraction of HBS is challenged by ramp‐like atrial signals and noise in PR segment of the cardiac cycle.

**Methods:**

By making use of a signal‐averaged trace showing prominent HBS as a guide trace, we developed a method combining interval‐dependent wavelet thresholding (IDWT) and signal space projection (SSP) technique to eliminate artifacts from single beats. The method was applied on MCG recorded on 21 subjects with known HV intervals based on EPS and noninvasive signal‐averaging, including five subjects with BSPM recorded subsequently. The method was also applied on stress‐MCG of a subject featuring autonomic dynamics.

**Results:**

HBS could be extracted from 19 out of 21 subjects by signal‐averaging whose timing differed from EPS between −8 and 11 ms as tested by 2 observers. HBS in single beats were seen as aligned patterns in inter‐beat contours and were appreciable in stress‐MCG and conspicuous than BSPM. The performance of the method was evaluated on simulated and measured MCG to be adequate if the signal‐to‐noise ratio was at least 20 dB.

**Conclusions:**

These results suggest the use of this method for noninvasive assessments on HBS.

## Graphical abstract

A novel method to unravel beat‐by‐beat surface recordings of His bundle signals is proposed. The method has been successfully demonstrated on 19 subjects for whom the invasive electrophysiological study had already been performed. This is inclusive of five subjects with BSPM measurements. The method has been qualitatively and quantitatively compared and these results persuade to use it for noninvasive assessments.

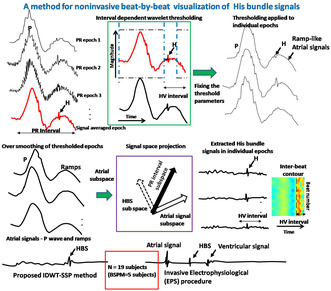

## 1 INTRODUCTION

The measurement of the time difference of the onsets of the spike‐like deflection of the His bundle (H) and the QRS complex (V) in the P wave‐to‐R wave interval (PR interval) of the cardiac cycle (Muresan et al., 2019) is a quantitative index of atrio‐ventricular conduction. Owing to the fact, the His bundle signals (HBS) are significantly weaker than the rest of the features of the cardiac cycle; its recording necessitates an invasive intra‐cardiac electrophysiological study (EPS) performed as a catheter‐based interventional procedure. HBS are bi or triphasic deflections ~100 Hz, and the HV interval is determined as the time difference between the onsets of the first wave after the intracardiac recordings of atrial signal and that of the surface recording of the Q wave seen in ECG. The normal range of HV interval is between 35 and 50 ms (Miller et al., 2018). A prolongation in the HV interval exceeding 75 ms hints at an eventual risk of interrupted electrical activation of the ventricles, needing implantation of a permanent artificial pacemaker to avoid complete heart block (Miller et al., 2018). Among the intracardiac signals and their time intervals that could be recorded in an EPS, HBS have a scope to be recorded by noninvasive methods by averaging several beats that are time marked to a chosen fiducial point on the cardiac cycle (Berbari et al., 1973; Flowers et al., 1974; Hombach et al., 1982; Vincent et al., 1978). HV interval is reasonably constant and normally maintains a temporal relationship with the QRS‐onset and is relatively unaffected by cardiac dynamics under normal conditions unlike other intervals of the cardiac cycle (Miller et al., 2018; Muresan et al., 2019). Hence, signal averaging procedure has been widely employed in investigations on noninvasive measurements of HBS in voltage‐based techniques such as the ECG/body surface potential mapping (BSPM) measurements (Berbari et al., 1973; Flowers et al., 1974; Hombach et al., 1982; Vincent et al., 1978) and in magnetocardiography (MCG), which records subtle cardiac magnetic fields (~100 femto Tesla–100 pico Tesla) (Tavarozzi et al., 2002). Among different magnetic field detectors and their varying configurations, MCG measured using superconducting quantum interference devices (SQUIDs) operating at 4.2 K temperature inside a magnetically shielded room (MSR) is regarded as a gold standard technique to record clinical MCG (Fenici et al., 1983; Sengottuvel et al., 2017; Senthilnathan et al., 2012; Tavarozzi et al., 2002; Yamada et al., 2003). MCG has been reported to offer complimentary diagnostic information on cardiac electrophysiology which is superior to that offered by voltage‐based measurements in a variety of cardiac dysfunctions (Tavarozzi et al., 2002). The list includes noninvasive measurement of highly resolved His‐signal features and HV intervals comparable to that of an EPS (Fenici et al., 1983; Sengottuvel et al., 2017; Senthilnathan et al., 2012; Yamada et al., 2003).

Biological artifacts such as very low‐frequency ramp‐like signals which are believed to be attributed to atrial repolarization in the PR interval undermine HBS in noninvasive measurements (Fenici et al., 1983; Sengottuvel et al., 2017) and further in retrieving them in a beat‐by‐beat manner. These low‐frequency signals are circumvented in invasive EPS measurements using filters with a typical bandwidth setting of 30–300 Hz (Muresan et al., 2019). Furthermore, HBS are reliably validated by moving the invasive measurement probe closer to the His bundle guided by X‐ray fluoroscopy in EPS. Because such maneuvers are not possible in noninvasive surface measurements, they are posed with an additional difficulty for rightly discriminating HBS from distortions and artifacts which are sometimes introduced by the signal conditioning methods itself (Feyter et al., 1981; Sengottuvel et al., 2017; ten Voorde et al., 1988). In order to unambiguously identify His bundle signals in a more reliable way, few researchers used multichannel ECG/MCG to seek additional information on the signal registrations of the His bundle by inspecting the spatial distribution patterns of signals obtained from iso‐field contour maps of multichannel data (Farrell et al., 1980; Fenici et al., 1983; ten Voorde et al., 1988). This suggestion had helped to better define HBS in noninvasive surface measurements and in determining the HV intervals comparable to invasive EPS traces (Sengottuvel et al., 2017). But that study could extract HBS only on signal‐averaged beats of the whole cardiac time series for each measurement channel (one representative beat per measurement channel), because of the use of signal averaging which helped to reduce inter‐beat noise. Recently, a technique on reducing beat‐by‐beat noise by treating the cardiac time series, as beat epochs was reported and was demonstrated to be helpful in the identification of MCG cardiac features based on inter‐beat similarities and time registries of cardiac events even under extremely noisy measurement conditions (Sengottuvel et al., 2021). It was intuitively persuading to employ a similar procedure for identifying HBS in every beat, by utilizing the relative constancy of HV interval on cases for whom HBS had already been identified based on multichannel ECG/MCG. The present work had drawn motivation from similar problems in neuroscience of recording event‐related potentials (ERP) in an electroencephalogram (EEG), by repeatedly presenting a specific stimulus to subjects and extracting the brain response pertaining only to the stimulus by trigger‐locked averaging (Luck, 2014). Quiroga and Garcia (2003) demonstrated that by using the trigger‐locked averaged ERP waveform as a guide trace, single‐trial brain responses could be retrieved by interval‐dependent wavelet thresholding (IDWT) by masking noise that occur other than the signal regime. Furthermore, the dynamics in neural responses were also visualized with the help of inter‐trial contour maps (Rey et al., 2015). The present work hence attempted to devise a method which would facilitate tracking HBS across beats by the conventional technique of averaging multiple beats to obtain HBS and use it as a template to define the signal regime for suppressing ramps and inter‐beat noise in the PR interval using IDWT. In addition, signal space projection (SSP) technique (Kunpeng et al., 2012) which is suitable to cancel common mode signal (i.e., ramp‐like variations) was employed in this work. Techniques on extracting HBS in every cardiac beat have not been reported so far in MCG and such studies in BSPM or ECG are also only sparingly discussed in the literature using proprietary hardware and software schemes (Ishijima, 1977;Liu et al., 2015; Wang et al., 2017). Moreover, the reported objective criteria in those ECG measurements involve inferring HBS from multiple micro‐wavelets in the PR interval necessitating subjective bias/practice in their identification (Liu et al., 2015; Wang et al., 2017). Hence, a signal treatment scheme is proposed on unambiguously validated HBS (by comparison with EPS), which were obtained by passively averaging individual MCG beats, and thereby, the proposed method has a scope to inspect their inter‐beat dynamics which were lost in the process of averaging. Thus, the method is expected to augment the visualization of HBS by tracking its components in a beat‐by‐beat manner in MCG and BSPM time series. Furthermore, the present work also demonstrates a possible application of the method in tracking HBS in an exercise MCG measurement where the beat‐by‐beat cardiac dynamics widely varied across the cardiac time series.

## 2 MATERIALS AND METHODS

### 2.1 Instrumentation and study protocol

The measurement data sets used in this investigation were recorded as a part of two independent studies (Kesavaraja et al., 2021; Sengottuvel et al., 2017) and were retrospectively analyzed for the present work. Institutional ethics committee approval and informed consent from subjects had been obtained for the original investigation. MCG data of 21 subjects (48 ± 11 years) were analyzed in this work. Out of these subjects (12 male, 9 female), two had ventricular tachycardia, and others had supraventricular tachycardia diagnosed based on ECG and EPS. The measurement of the HV interval formed a part of the usual detailed evaluation in EPS. The EPS was measured following the conventional clinical procedure in a hospital setting. MCG measurements were done in a different institution by an independent research group on different days. All these subjects were hemodynamically stable and were accompanied by a cardiologist when brought for MCG measurements. For five of these subjects, BSPM was recorded subsequently after MCG measurements. Stress‐MCG was measured from a healthy control (M33) involved in physical exertion using a non‐magnetic bicycle ergometer attached to the MCG system.

MCG was measured using a thirty‐seven‐channel SQUID system operating inside a low noise environment facilitated by a magnetically shielded room (MSR) (Parasakthi et al., 2012). The room contained two layers of mu‐metal and two layers of Aluminium capable of attenuating external electromagnetic interferences with a shielding factor better than 100 dB at 100 Hz, 70 dB at 1 Hz and 50 dB at 0.1 Hz. The MCG measurement system consisted of a hexagonal array of low transition temperature, direct current biased SQUID sensors (Parasakthi et al., 2012). The sensors were housed in a flat‐bottomed liquid Helium cryostat made of fiber reinforced plastic. The warm‐to‐cold distance of the cryostat was 1.5 cm which dictated the minimum most distance of separation between the chest of subjects and the sensor plane. The SQUID electronics was operated with a bandwidth setting of 0–300 Hz. The subjects were positioned in supine posture with the chest facing the bottom tail of the suspended cryostat mounted on a non‐magnetic gantry. The chest of the subjects was aligned under the cryostat about anatomical landmarks such as the collar bone, sternal line, fourth intercostal space, and xiphoid process. The average noise floor measured across the 37 channels in the MCG system was 25 ± 9 *fT*_rms_/√Hz. For the healthy control subject involved in stress measurement, the MCG measurement was made during a pedaling action in supine posture in the ergometer, creating a moderate stress condition (Kesavaraja et al., 2021).

BSPM measurements were made using a sixty‐four‐channel EEG system (Compumedics Ltd.) which can also function as a general‐purpose bio‐potential recorder (Patel et al., 2016). The amplifier was set for a gain of 1000, bandwidth set at 0–1000 Hz (and later band‐limited to 300 Hz). The digitization accuracy of the system was 30 nV per least significant bit, and the peak‐peak noise amplitude of the system was <1 μV_pp_. BSPM measurements were made precisely on the same thirty‐seven locations on the chest as measured by the MCG system. Discrete Ag–AgCl electrodes were used to measure the potential difference across the chest locations. The signal processing methods and their sequence were the same for both MCG and BSPM cardiac time series.

### 2.2 Method for beat‐by‐beat visualization of His bundle signals

#### 2.2.1 Presentation of MCG/BSPM time series

**FIGURE 1:**
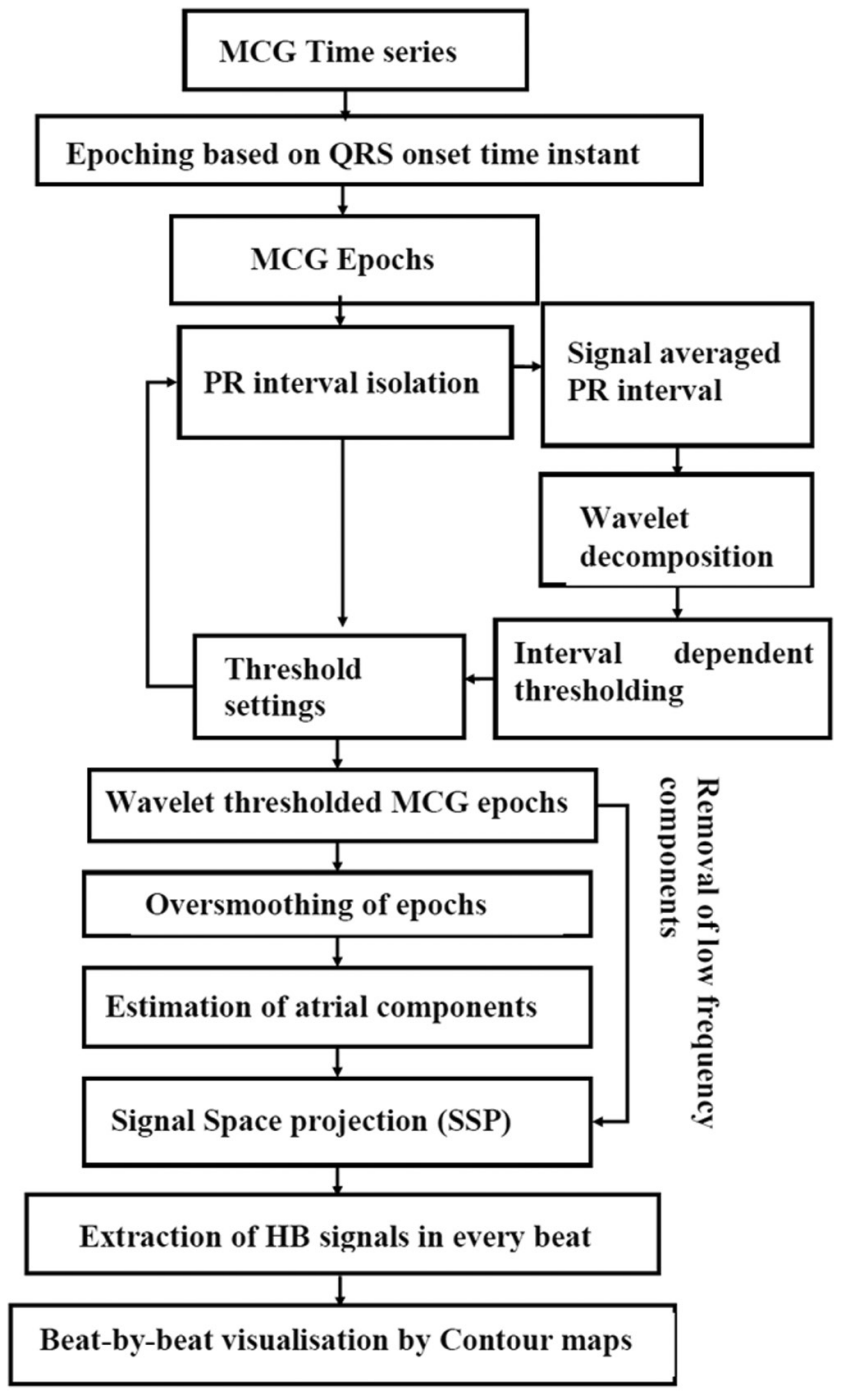
Block diagram of the proposed beat‐by‐beat analysis method for the visualization of His bundle signals.

Figure 1 illustrates the schematic of the proposed method. The MCG/BSPM time series of a channel with the least noise floor was chosen for analysis in each case. The cardiac time series which was recorded for 3 min was epoched and stacked with respect to the time point (sample number) of the onset of the QRS by an automated approach (Sengottuvel et al., 2017) such that at least 200 consecutive cardiac cycles (beats) are taken for further analysis. The PR intervals of all 200 cardiac beats were isolated and averaged to get one representative signal‐averaged trace. Each of these averaged waveforms showed a P wave followed by ramp‐like slowly varying signals, a small bump and again ramps up to the QRS‐onset in all the measurement channels but with differing magnitudes and configurations across spatial locations. The objective criteria followed in this work to define a bump (or a deflection) in the signal‐averaged PR interval waveforms as the His bundle signal are as follows:

- The chosen deflection in the PR segment was above the noise floor that was observed before the onset of the P wave, where no cardiac activity could be expected.
- The chosen bump was ensured to be well delineated from the offset of the P wave observing its time registries in all the channels.
- The chosen bump/deflection was surrounded by ramp‐like signals before and after its occurrence.
- The later part of the ramp that followed a chosen bump changed its polarity across the measurement channels, i.e., that which changed from a linearly increasing to decreasing variation and vice‐versa.

**FIGURE 2:**
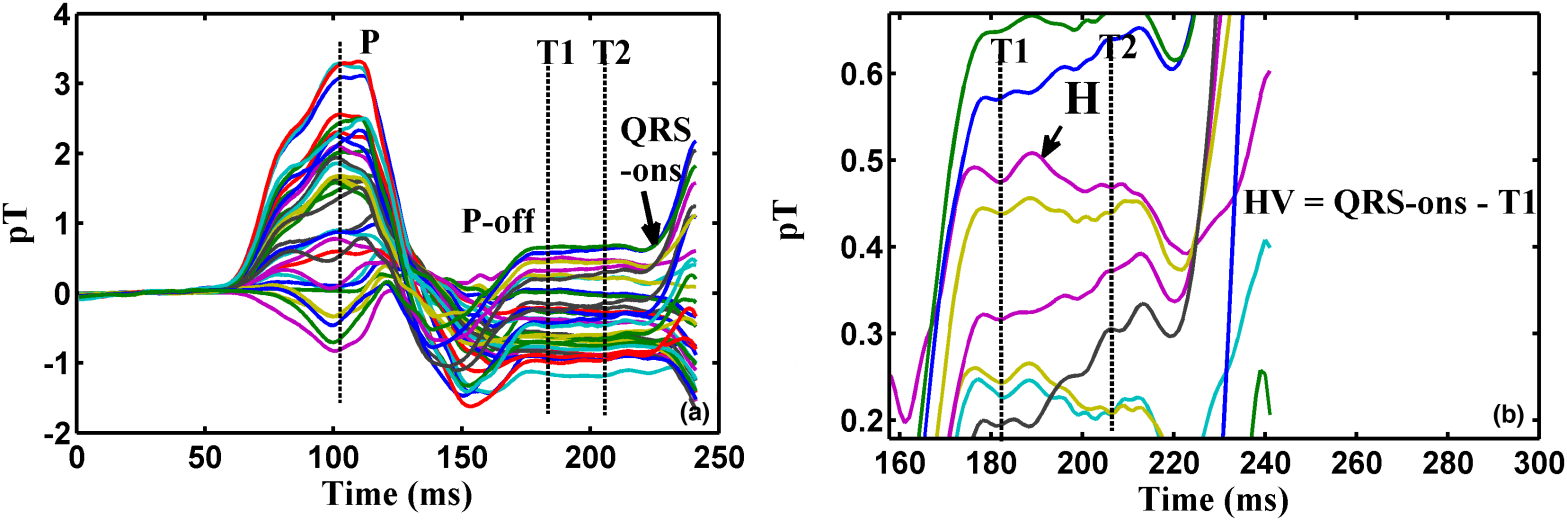
Identification of His bundle signals from overlay plot of signal‐averaged MCG waveforms across 37 measurement locations (each colored line representing MCG measurement channel) (a) PR interval of signal‐averaged MCG waveforms showing cardiac deflections of interest namely P wave, offset of P wave and QRS‐onset. Time instants T1 and T2 are manually marked, respectively, after P wave offset and before QRS‐onset on ramp‐like signals encompassing a bump (b) Magnified view of (a) showing a bump attributed to HBS (H) and the ramp marked at T2 changes polarity across measurement locations. HV interval computed as the difference between the QRS‐onset and T1 is also indicted.

This physiological sequence of events in the PR interval was well‐studied by researchers (Fenici et al., 1983; ten Voorde et al., 1988) and was utilized to identify HBS in an earlier work (Sengottuvel et al., 2017). The ramp‐like signals were reported to be attributed to atrial repolarization which was known to be dominant in the initial portion of the PR interval and progressively subdued at the later part after His bundle deflection (Fenici et al., 1983). The objective criteria and these descriptions could be comprehended better by plotting an overlay plot of the signal‐averaged waveforms as illustrated in Figure 2. The colored traces in the figure represent thirty‐seven signal‐averaged waveforms of the MCG measurement channels and the cardiac time series of each channel is epoched, averaged by stacking 200 cardiac beats. The averaged waveforms of all thirty‐seven channels are superposed as shown in Figure 2a to infer the time registry of events that occur in the PR interval across measurement locations. The overlay plot thus helped to better visualize the time instant at which the ramps change their polarity as required to test the objective criteria. As could be seen in the magnified view of the PR interval in Figure 2b, a bump‐like deflection rides over ramps in the waveforms of all the channels. Two vertical lines (T1 and T2) were manually drawn based on visual inspection such that they were placed before and after a chosen bump in the PR segment to identify the physiological origin of the ramps under discussion. As could be noticed in the figure, the ramps at time instant T2 changes its polarity across measurement channels and thus fulfills the criteria to define the chosen bump as HBS. The identified HBS from signal‐averaged traces in MCG and their respective HV intervals which were noninvasively deduced were verified at a later stage with their corresponding EPS report in a blind‐folded manner by two independent researchers after MCG measurements and their analysis were completed. Detailed descriptions of this analysis are given in an earlier work (Sengottuvel et al., 2017). Furthermore, the HBS and the HV intervals of a subset of cases used in this analysis were revalidated in an unbiased manner by two electrophysiologists as independent observers who were unaware of the proposed method. The two observers were requested to identify HBS and to determine HV intervals in the PR interval and the values were compared against the HV intervals determined already by us as a reference set. The morphology of the obtained HBS in MCG varied from a sharp spike (“M” shaped biphasic deflection) to a bump, riding over the ramps. In some cases, the bumps were superposed with high‐frequency wiggles which were attributed to myoelectric noise or other random fluctuations in the PR interval (Hombach et al., 1982; Yamada et al., 2003). The signal‐averaged MCG trace with verified HBS was then subjected to interval dependent wavelet thresholding and signal space projection technique. The mathematical basics of IDWT and SSP are given elsewhere (Donoho, 1995; Uusitalo & Ilmoniemi, 1997) the following details describe the relevance and the way of implementation of these techniques in the present work to extract HBS in every beat.

#### 2.2.2 Wavelet thresholding

Wavelet thresholding aimed to reduce the background signals present in the PR interval other than the time segment of HBS (in the HV interval). Wavelet Toolbox of MATLAB software, version R2013a (The MathWorks Inc.) was used for IDWT. The averaged signal was decomposed to a set of approximation and detail coefficients by ‘coif 5’ wavelet in five levels of decomposition. The choice of this wavelet basis was based on a report which discussed its suitability for analyzing His bundle signal features (Lee, 2004). The magnitude values of each of the detail coefficients were thresholded by fixed form soft thresholding by keeping the time intervals around the chosen HBS unaffected (few milliseconds of time points on both sides of the HBS bump or deflection) by manually setting the threshold intervals (Donoho, 1995). The number of intervals was fixed based on the discernibility of HBS overlapping with the preceding and succeeding ramps in the PR interval. For the cardiac signals used in the present work, either 4 or 5‐time intervals were found to be sufficient for IDWT. The chosen time intervals with the set of threshold parameters were stored as a function and applied on all the individual raw epochs for a given subject. Because thresholding technique (Donoho, 1995) could be performed only on the detail coefficients, the attenuation of the low‐frequency ramps which fell outside the chosen region of interest (within the HV interval) was taken care of separately by the SSP technique.

#### 2.2.3 Signal space projection technique

SSP aimed at separating HBS from the PR interval by creating an orthonormal basis function of the atrial components generated from the PR epochs. The wavelet thresholded epochs of the PR intervals were smoothed by applying Savitzky–Golay (SG) filter which acted as a local least square polynomial estimator (Hargittai, 2005). The order of the SG filter was set as 3 (cubic) with a frame length of 23 to be suitable for over‐smoothing operations (Krishnan & Seelamantula, 2013). Since the HBS are high frequency signals, the smoothed epochs were expected to contain only atrial components: the P wave and ramps in the PR interval.

The measured signal in the PR interval is a combination of physiological events of the HBS and the atrial activity (comprising depolarization and repolarization of atria) as represented by Equation (1). It is reasonable and physiologically consistent to assume that both signals are from different sources (Berbari et al., 1973; Flowers et al., 1974; ten Voorde et al., 1988; Vincent et al., 1978).

(1) $$b_{\mathrm{PR}}\left(t\right)=b_{\mathrm{HB}}\left(t\right)+b_{A}\left(t\right)$$

If *b*_A_(*t*) was characterized by magnetic field/electric potential waveforms of ‘m’ number of epochs of a chosen measurement channel, then a matrix *U*_n_ with each column forming an orthonormal basis vector could be constituted by singular value decomposition (Uusitalo & Ilmoniemi, 1997). The vector space spanned by *U*_n_ was designated as the atrial subspace. The size of *U*_n_ was less than ‘m’ comprising only significant basis vectors, which was sufficient to represent *b*_A_(*t*). It was then possible to form a subspace *P*_per_ that was orthogonal to the atrial subspace and was devoid of atrial components as given by Equation (2).

(2) $$P_{\mathrm{per}}=I-U_{n}U_{n}T$$

where, *I* is an identity matrix of the same size as *U*_n_.

By projecting the measured signal over this new subspace *P*_per,_ the His bundle signals could be selectively retrieved from the PR interval epochs as given by Equation (3).

(3) $$b_{\mathrm{HB}}\left(t\right)=P_{\mathrm{per}}\times b_{\mathrm{PR}}\left(t\right)$$

Detailed descriptions of SSP technique and its use for removing common‐mode background artifacts are given elsewhere (Sriram et al., 2013).

The extracted HBS were visualized by inter‐beat contour maps generated throughout the MCG epochs of 200 consecutive beats. However, for better visibility of the HBS, the beat contours are illustrated in this work for a time segment of length ~25 beats.

### 2.3 Evaluation of the proposed analysis method

#### 2.3.1 Simulation study on the proposed method in retrieving HBS

The proposed analysis method was assessed for its ability to extract HBS under varying levels of atrial influence and random noise using a synthetic signal representing a PR interval epoch containing a reference HBS at an interval of 60 ms before the QRS‐onset (i.e, HV = 60 ms). Varying magnitudes and configurations of ramps mimicking atrial repolarization activity and fluctuations caused by random noise which corrupt the PR interval were simulated. This simulation involved generating three different types of atrial T wave (PT_a_) (atrial repolarization wave) ramps that commonly occur in the PR interval (Farrell et al., 1980; Jayaraman et al., 2016). Atrial repolarization waveform with its magnitude one‐third of that of the P wave, but of opposite polarity (designated as Type 1), PT_a_ with magnitude one‐half of that of the P wave (designated as Type 2), and PT_a_ with a magnitude exceeding one‐half of the magnitude of P wave in the positive direction (designated as Type 3) were used. Additive white Gaussian noise was added to each type of the simulated PR interval waveform with signal‐to‐noise ratio (SNR) ranging between 5–28 dB (with P wave peak regarded as the signal magnitude). Pearson's correlation coefficient was calculated between a reference HBS (containing only the HBS without random noise and atrial components) and the retrieved HBS using the proposed method in each type of the simulated waveform.

#### 2.3.2 Quantitative parameters computed from the retrieved HBS in the measured MCG

Two quantitative parameters namely signal‐to‐noise ratio (SNR) and signal‐to‐error ratio (SER) (Rajesh et al., 2017), the former in time and the latter in frequency domain were used to evaluate the efficiency of the proposed method in reducing the atrial activity and random noise. SNR values were calculated at P wave peak and that of extracted HBS against the noise present before the onset of the P wave in the PR interval of a signal‐averaged beat. These values were compared against those calculated for one of the beat epochs in which the HBS were extracted using the proposed IDWT‐SSP method. SER was computed using the following Equation (4).

(4) $$\mathrm{SER}=\frac{\text{Power of extracted signal}}{\left(\text{Power of}\;\mathrm{raw}\;\text{signal}-\text{Power of extracted signal}\right)}$$

SER was expected to be higher at the frequency range of signals signifying the preservation of the content of the signal and was expected to be least when calculated at unwanted noise frequencies indicating their envisaged reduction by the de‐noising methods (Rajesh et al., 2017). SER was computed from the power spectral density at the frequency range of P wave (5–30 Hz), HBS (75–100 Hz) and noise (150–200 Hz). These discrete frequency regimes were fixed based on the frequency spectrum of signal‐averaged waveforms of PR intervals showing the frequency components of P, HBS and noise for all the subjects discussed in this study. The frequency regime 150–200 Hz always contained noise components and were found to be less likely to contain signal features of interest in this study. Hence, the exclusive contribution of the methods used in this study to the reduction of random noise was evaluated at this noise frequency range.

### 2.4 Statistical analysis on inter‐observer variability of HV intervals

By treating the HV interval values determined already by us as a reference set, the values calculated by the two independent electrophysiologists were compared by computing Spearman's rank correlation coefficient (*ρ*) which is widely used to assess inter‐observer variability in medical research (Patrick et al., 2018) as given by this following equation.

(5) $$\rho =1-\frac{6\sum d_{i}^{2}}{n\left(n^{2}-1\right)}$$

where *d* is the difference between the two ranks of HV intervals measured by each observer, and *n* is the number of HV interval measurements.

In addition, Bland–Altman analysis (Giavarina, 2015) was also performed on the differences of HV intervals and those calculated by the two independent observers to evaluate their level of agreements.

## 3 RESULTS

### 3.1 Results on the application of the proposed method on cardiac time series

**FIGURE 3:**
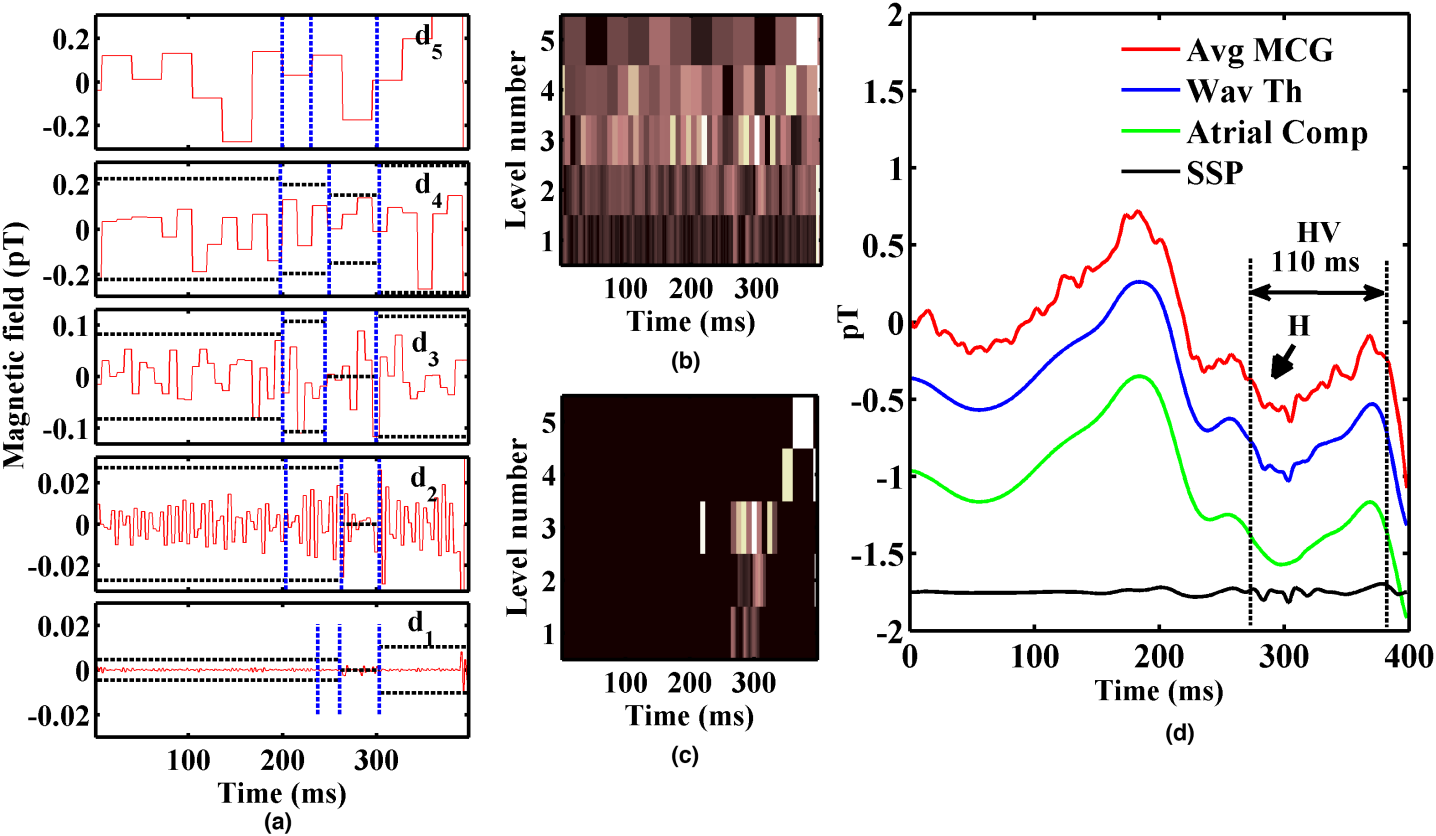
Stage‐by‐stage outputs of the proposed method applied to the MCG measured on a subject with HV interval 120 ms (a) Detail coefficients (d1–d5) of a signal‐averaged PR interval of MCG trace obtained by wavelet decomposition. The chosen time intervals (blue vertical dotted lines) and their magnitude limits (black horizontal dotted lines) for the thresholding process are also marked on the detail coefficients, (b) Wavelet coefficient plot at different levels of decomposition for the raw and (c) thresholded coefficients, (d) Traces in the order from top to bottom. Raw averaged MCG trace of PR interval (red), wavelet thresholded trace (blue), over‐smoothed PR interval comprising P wave and ramps in the PR interval (green), extracted HBS after SSP back projection (black). The traces in (d) are baseline shifted for clarity.

**FIGURE 4:**
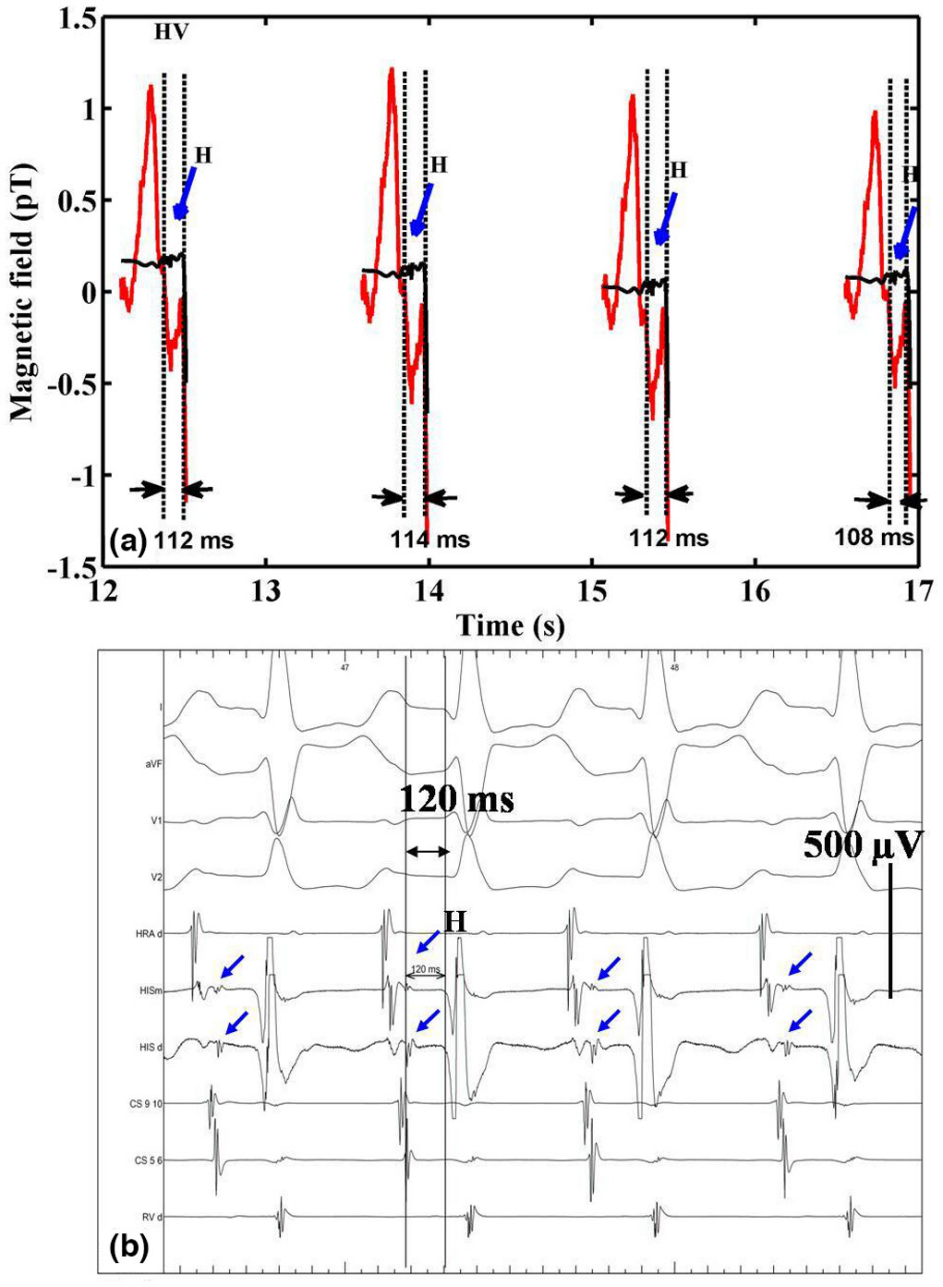
(a) Raw PR intervals of a chosen time segment of MCG superposed with extracted HBS using the proposed method. The HV intervals determined are also marked in every beat. (b) The corresponding invasive EPS recordings featuring HBS spike (indicated by arrows) marked with the measured HV interval of 120 ms.

The stage‐by‐stage outputs of the proposed analysis method comprising IDWT and SSP are shown in Figure 3. A discernible HBS seen in the averaged PR interval trace was taken as a template for IDWT. HBS in MCG (defined with its onset) was identified based on the criteria followed in an earlier work with respect to multichannel information (Sengottuvel et al., 2017). The HV interval measured as 110 ms for this subject in MCG was comparable to that determined in intracardiac recordings as 120 ms. Five detail coefficients of the template epoch obtained by subjecting the signal trace for wavelet decomposition with five levels are shown in the figure. The detail coefficients, d4, and d5 do not represent HBS since they contain only very‐low‐frequency components and were fully thresholded as shown in the figure. Since the HBS occurred around the time interval 270–310 ms, the detail coefficients other than this time segment were thresholded in d1–d3 by manually fixing the time limits. The plot on wavelet coefficients across the decomposition levels for the raw and the thresholded ones are shown in Figure 3b,c. The thresholding process had preserved the HBS features while removing other noisy wiggles outside this region, as seen in the thresholded output waveform in Figure 3c and is evident in the wavelet thresholded waveform shown in Figure 3d (blue trace). Still, the ramp‐like variations before and after the HBS are unaffected by IDWT. The over‐smoothing process performed on the IDWT trace contained only the P wave and ramps (green trace) and whose back‐projection resulted in isolation of high‐frequency HBS as shown in the black trace in Figure 3d. The thresholding process with the set parameters for the template epoch were stored and applied on the individual PR interval epochs of all the raw individual beats of the cardiac time series. The extracted signals from each beat of a measurement MCG channel of this subject are illustrated in Figure 4 as MCG time series along with the raw epochs in their respective beat order and compared with the EPS recording of the same subject. Minor variations in the measurement of beat‐by‐beat HV intervals do exist and are indicated in the figure.

**FIGURE 5:**
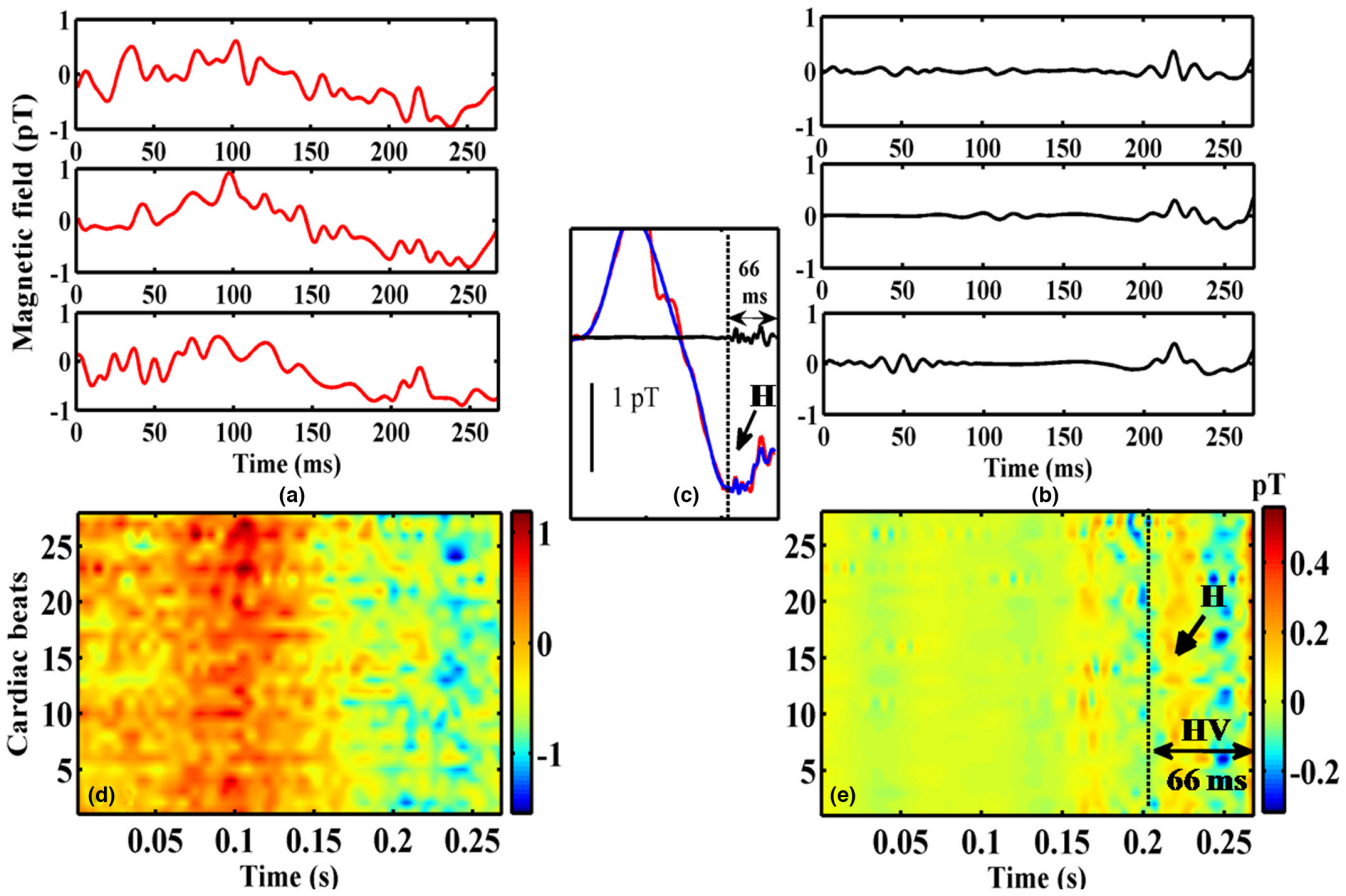
Visualization of HBS in inter‐beat contour map (a) input raw PR interval of three representative epochs and (b) their output waveforms applying the proposed method (c) signal averaging of all the PR epochs (red), wavelet thresholding applied on the averaged trace (blue), extraction of HBS after SSP (black) (d) inter‐beat contour map of ~25 beats generated on the raw epochs and (e) generated on the extracted epochs selectively highlighting the HBS.

**FIGURE 6:**
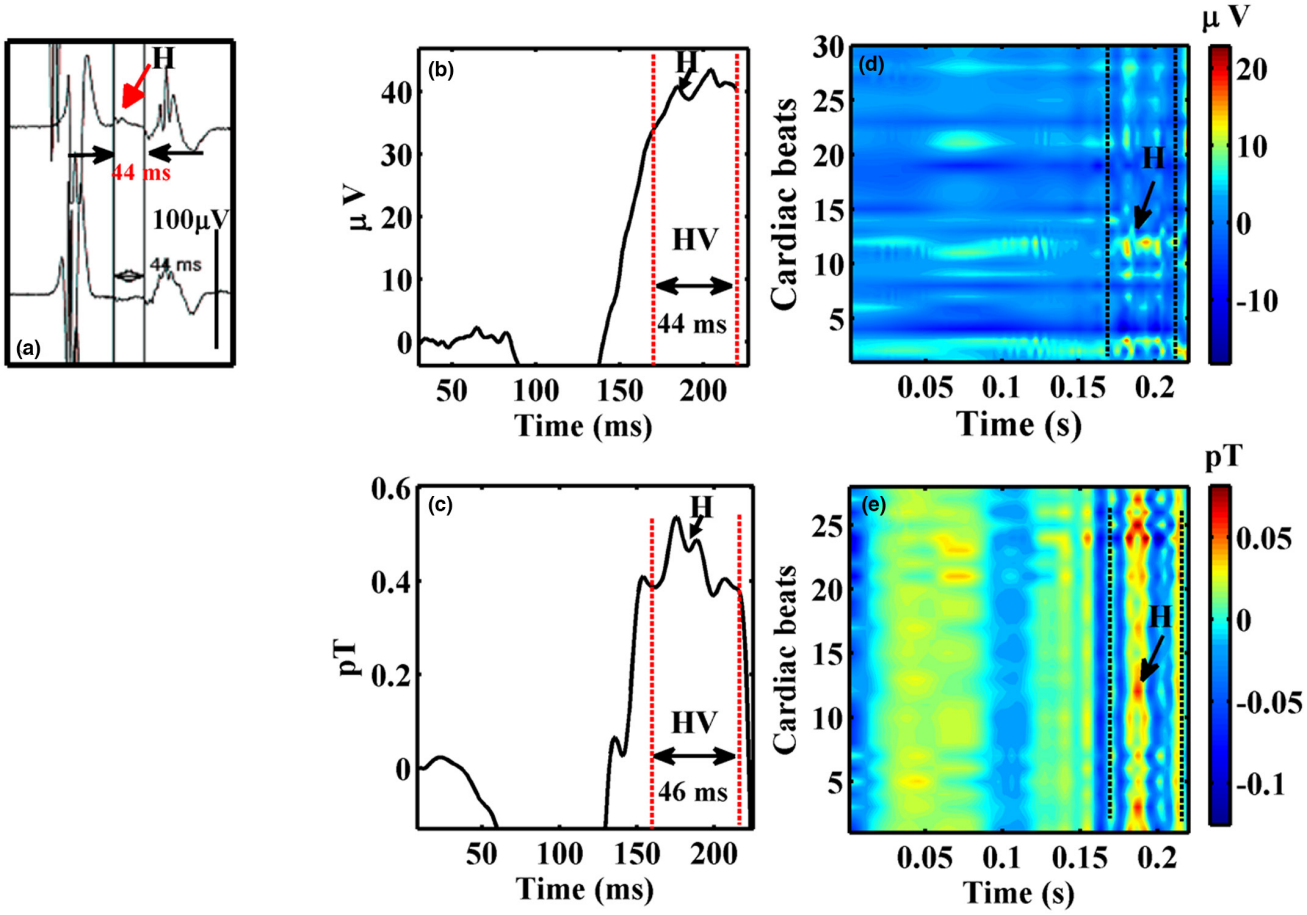
Comparison of HBS features of a subject with HV interval 44 ms in (a) EPS, (b) signal‐averaged BSPM, (c) signal‐averaged MCG, and their respective inter‐beat contour maps (d) and (e).

**FIGURE 7:**
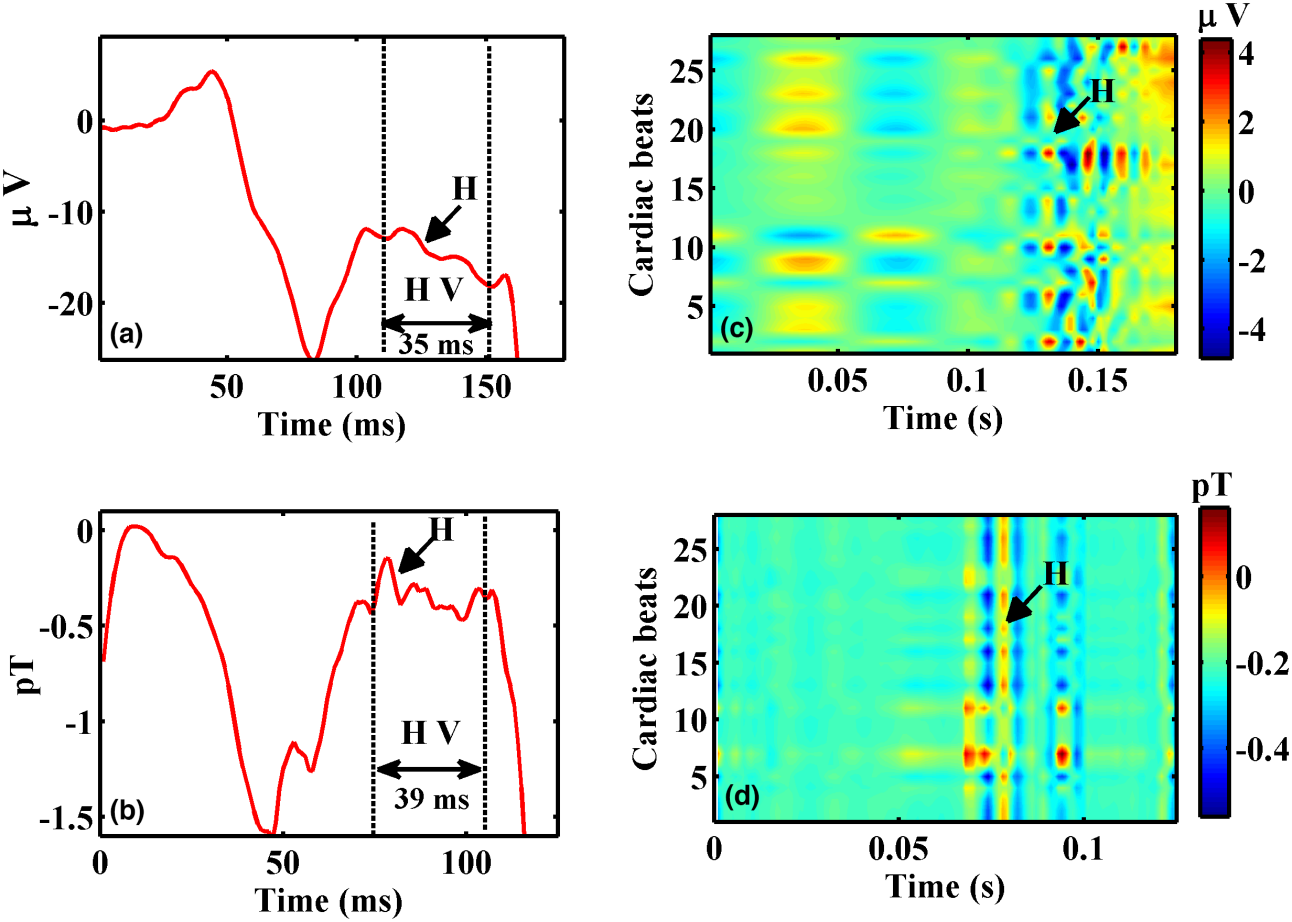
HBS visualized in averaged traces of (a) BSPM and (b) MCG and their inter‐beat contour maps in (c) and (d). His bundle signals are indicated by arrows.

Figure 5 shows another example of a subject with HV 70 ms based on intracardiac recordings (66 ms in the noninvasive MCG). Three representative noisy raw epochs of the PR interval of MCG are shown in Figure 5a, and the application of this method which selectively isolated HBS from the raw epochs, is shown in Figure 5b. It is to be noted in Figure 5c, the conventional method of averaging these epochs could retrieve HBS (red trace in Figure 5c) by attenuating uncorrelated noise across the epochs. Wavelet thresholding significantly augmented the visualization of HBS by eliminating noisy wiggles outside the region of HBS (blue trace in Figure 5c). The qualitative improvement in the extracted HBS by IDWT and SSP are comprehended better in the inter‐beat contour maps shown in Figure 5d,e. It could be observed that the higher magnitude atrial components and noisy wiggles seen in the contour map of the raw epochs Figure 5d were drastically reduced by the proposed method and the HBS are appreciably aligned as colored patches within the HV interval and aid a direct visualization of HBS almost in every beat as shown in Figure 5e. In all the five subjects, the BSPM and MCG exhibited HBS with mean difference in HV intervals between the two modalities less than 3 ms. Figures 6 and 7 depict the results obtained from two representative cases with HV intervals 44 and 37 ms. The extracted HBS features in MCG for both these cases were found to be resolved than that seen in their BSPM recordings. In both the figures, measurement channels in which the cardiac features as well as HBS which were morphologically similar in MCG and BSPM in the measurement locations on the chest which were closer are chosen for this qualitative comparison. The HBS obtained in MCG exhibited its characteristic double hump morphology (‘M’ shaped) (Senthilnathan et al., 2012) and were traceable throughout the time length. These observations on HBS were markedly different in BSPM which are seen only as sporadic and smeared out traces in their corresponding inter‐beat contours for both cases in Figures 6 and 7.

### 3.2 Results on the quantitative evaluation of the proposed method

**FIGURE 8:**
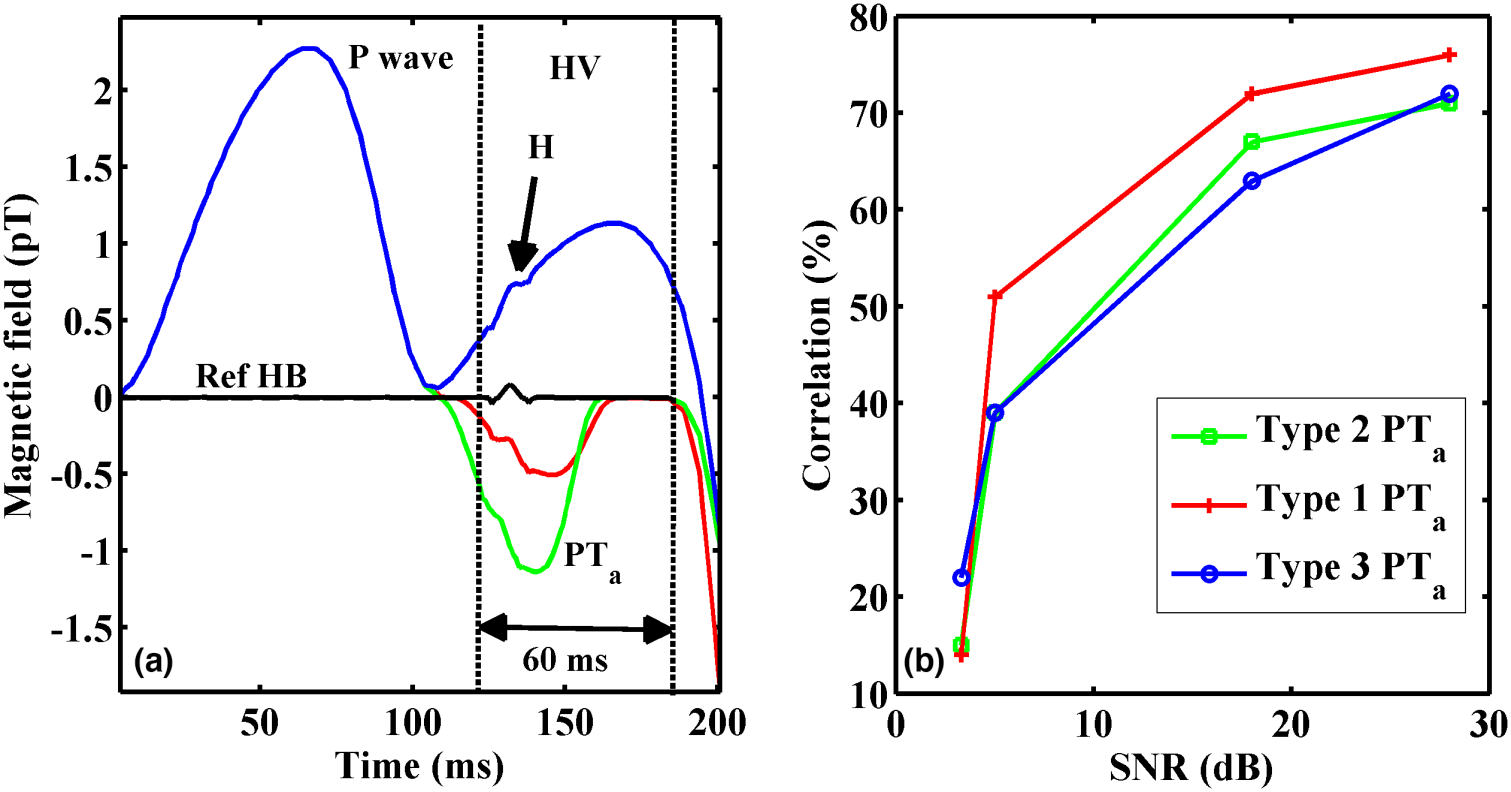
(a) Performance of the proposed method in retrieving HBS on simulated PR intervals with three different types of atrial repolarization (PT_a_) waveforms. Reference HBS is also shown (black trace), the onset of first downward deflection of HBS is taken as its onset (b) Correlation between the extracted and reference HBS calculated at different signal‐to‐noise ratios for the three types of atrial repolarization waveforms.

Figure 8a shows simulated PR epochs containing HBS masked by three different types of PTa waves. The performance evaluation of the proposed method is represented in Figure 8b as a plot between the correlations of extracted HBS against SNR of the simulated signal. The plot indicates that the HBS could be extracted better when the SNR was above 25 dB, based on the correlation value taken as an index and was maximum for Type 1 atrial repolarization compared to the other two types of PT_a_. Table 1 presents the SNR and SER values calculated from the signal‐averaged PR interval of MCG trace and HBS extracted using the proposed IDWT‐SSP in one of the epochs for all the subjects.

**TABLE 1:** Quantitative assessment on the proposed method.

Sl.no	SNR (dB)		SER			Method
	P wave	HBS	P wave	HBS	Noise	
1.	18	12	22	0.3	0.08	SAMCG
	4	18	0.004	1.06	0.004	IDWT‐SSP
2.	18	12	5	0.02	0.01	SAMCG
	1	9	8E‐5	0.82	1E‐5	IDWT‐SSP
3.	20	1	12	0.01	0.002	SAMCG
	1	6	0.003	2.1	3E‐5	IDWT‐SSP
4.	18	3	15	1.3	0.4	SAMCG
	6	13	1E‐4	2.35	2E‐7	IDWT‐SSP
5.	15	8	25	0.6	0.01	SAMCG
	2	21	6E‐5	13.5	6E‐4	IDWT‐SSP
6.	10	1	22	0.07	0.003	SAMCG
	1	16	2E‐4	7.8	5E‐7	IDWT‐SSP
7.	34	15	20	0.07	1E‐3	SAMCG
	3	19	0.043	5.9	6E‐5	IDWT‐SSP
8.	29	10	29	3.1	3E‐3	SAMCG
	2	31	1E‐5	15	2E‐6	IDWT‐SSP
9.	30	6	18	0.03	1E‐4	SAMCG
	1	7	1E‐4	1.8	2E‐5	IDWT‐SSP
10.	29	25	3.6	0.1	4E‐3	SAMCG
	2	9	0.002	3.52	7E‐4	IDWT‐SSP
11.	21	18	36	0.8	2E‐4	SAMCG
	2	10	4E‐3	5.38	8E‐5	IDWT‐SSP
12.	16	2	14	0.2	3E‐3	SAMCG
	3	1	0.009	2.45	2E‐6	IDWT‐SSP
13.	29	6	26	0.76	0.04	SAMCG
	4	12	0.6	6.6	2E‐4	IDWT‐SSP
14.	12	9	17	2.4	2E‐4	SAMCG
	2	4	8E‐4	8.11	6E‐5	IDWT‐SSP
15.	19	11	5.4	0.1	3E‐4	SAMCG
	1	7	1E‐4	3.4	2E‐5	IDWT‐SSP
16.	30	8	51	0.5	1E‐3	SAMCG
	2	16	7E‐6	1.09	3E‐6	IDWT‐SSP
17.	14	10	13.1	0.67	5E‐3	SAMCG
	6	5	1.2E‐4	5.4	1E‐5	IDWT‐SSP
18.	38	6	10.23	2.4	0.02	SAMCG
	5	3	5E‐3	1.48	1E‐3	IDWT‐SSP
19.	18	9.6	22	4.3	1E‐4	SAMCG
	4	7	9E‐4	6.5	5E‐5	IDWT‐SSP

Although MCG data of 21 subjects were analyzed in this study, single beat extraction of HBS could be achieved only in 19/21 subjects using the proposed method due to very high level of atrial ramps in the other two subjects. It could be noticed from the Table that SNR of the as‐presented signal‐averaged MCG trace was already adequate (~15–20 dB) for HBS, but with significant dominance of atrial activity seen from SNR at P wave. This was drastically reduced by the proposed method even in a single cardiac beat. Similarly, the observations of higher values of SER at HBS frequency and low SER values at P wave and at noise frequencies further endorsed the fact the prominence of HBS alone was facilitated in the PR interval by the proposed IDWT‐SSP method.

### 3.3 Results on the application of the method to stress MCG

**FIGURE 9:**
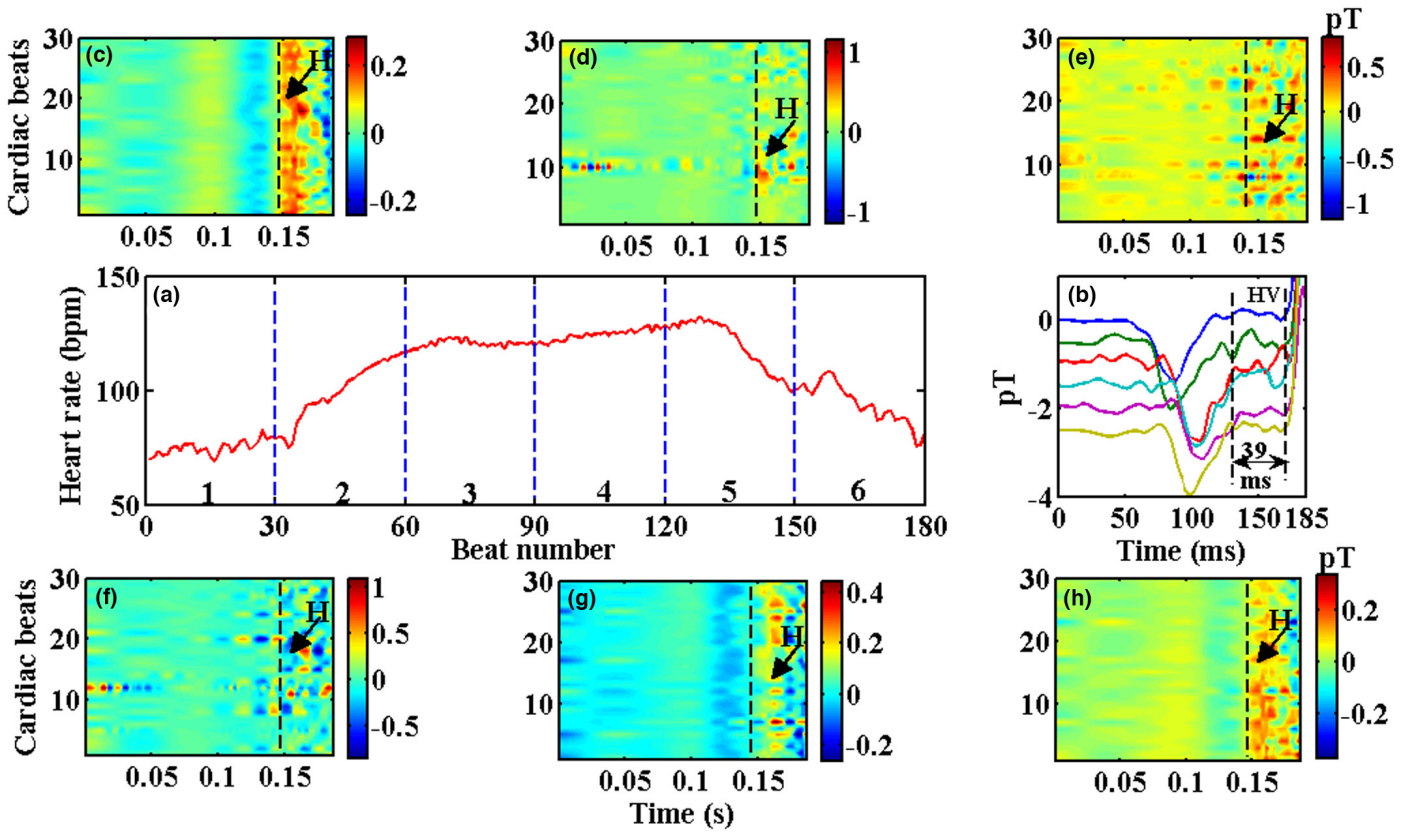
Performance of the proposed method in stress MCG (a) Heart rate variations exhibiting a large extent of autonomic variations across the beat numbers, demarcated by analysis bins of 30 beats each (b) Signal‐averaged beats of each bin are shown in the order top to bottom trace. (c–h) Inter‐beat contours of the bins (1)–(6) using the proposed method. Arrows indicate traceable HBS in the contours.

Figure 9 features the application of the proposed method to MCG measured on a healthy subject during a moderate stress condition, characterized by beat‐by‐beat autonomic variations in the heart rate. Figure 9a shows heart rate variations plotted against the beat numbers. A gradual increase in the heart rate commensurate with physical stress causing fluctuations from a normal resting state ~80 beats per minute (bpm) to a maximum of 120 bpm and back to ~80 bpm is shown. The beat numbers (epochs) were classified into six analysis bins each of 30 beats in length. Signal‐averaged epochs (of PR interval) in each analysis bin are shown in Figure 9b in the order from top to bottom (representing bins 1 to 6). The averaged traces show an inverted P wave with short PR segments (the portion of the PR interval after the P wave offset) with bumps attributed to HBS identified based on criteria described earlier with reference to signal registration in multiple channels (Sengottuvel et al., 2017). Due to the relative constancy of HV interval irrespective of the autonomic variations, the averaged traces show the portion of the PR segment containing the bump (H) to be temporally stable about the QRS‐onset (~39 ms). The phase shift in the P waves of the averaged traces was attributed to the cardiac dynamics imposed by autonomic influences (Senthilnathan et al., 2012). The fall in SNR of the averaged traces in the analysis bins 2, 3, 4, and 5 was due to the significant influence of heart rate variations and the measurement noise imposed by the physical action of the subject. The contour maps generated on each analysis bin using the proposed method for extracting HBS are shown in Figure 9c–h. Consistent with the observation on the averaged traces of each bin, the contour maps show the HBS as red aligned patches which are easily traceable except for Figure 9e,f, where the noise levels are too high, limiting the visibility of HBS using the proposed method. The visualization provided by this method highlighted the possibility of beat‐by‐beat analysis of His bundle signals (as ordered features), especially in Figure 9c,h.

### 3.4 Results on the statistical analysis on HV interval

**FIGURE 10:**
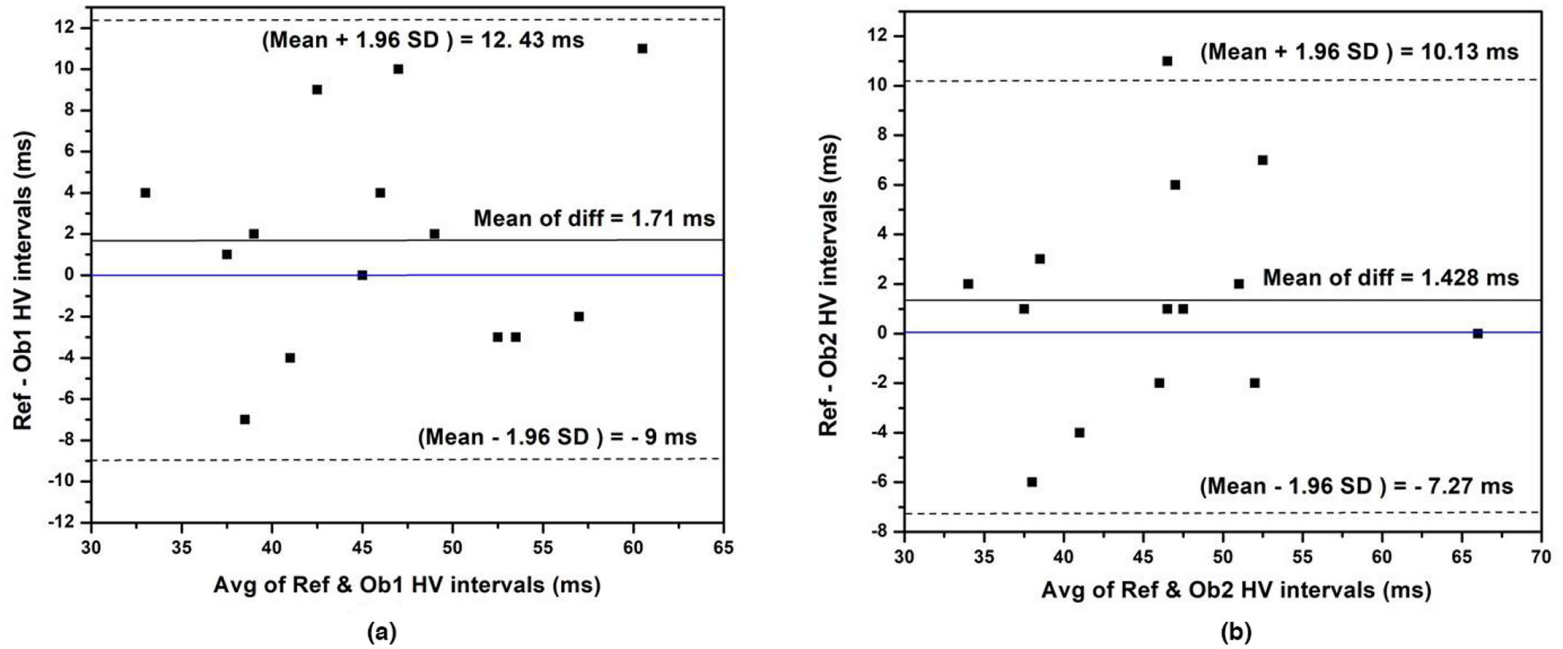
Bland–Altman plot of inter‐observer variations on HV intervals against reference (Ref) measurements (a) Observer 1 (Ob1) and (b) Observer 2 (Ob2).

The Spearman's rank correlation coefficient (ρ) on the inter‐observer variability of HV intervals was computed using Equation (5) to be 0.7966 for observer 1 and 0.776 for observer 2, and their agreements with our reference measurement were statistically significant with *p* value <.00065 for both the cases. Figure 10 shows the plot on Bland–Altman analysis performed on the differences of HV intervals by the two independent observers. As could be seen from the figure, the mean differences are 1.7 and 1.43 ms, respectively, for observer 1 and 2. The upper and lower limits of agreement at ±1.96 times standard deviation are calculated as −9 to 12 ms for observer 1 and −7 to 10 ms for observer 2. Hence, the overall mean of the limits of agreement is −8 to 11 ms, and these figures indicate that there is no significant difference in HV values across the observers, validating the analysis presented here on further extracting the HBS in a beat‐by‐beat manner.

## 4 DISCUSSION

The present work reported the surface registration of the HBS using MCG in a beat‐by‐beat manner by logically approaching the problem of HBS tracking, utilizing the relative constancy of HV interval. In the absence of any clue on the signal features in the individual beats, traces of signal components that eventually contribute to the overall evolution of the averaged HBS components were visualized using the proposed method. Even though the number of cases shown in this work was only a few, the formulation of the proposed approach was more viable owing to their corroboration with invasive EPS for all the cases studied and as shown in Figure 4. The fact that His bundle signals and HV intervals were revalidated by two independent observers and supported by the statistical analysis, they summarily suggest an overall agreement on the HBS and their HV intervals justifying the analysis proposed in the manuscript to be taken as an unbiased method.

Second, comparison of BSPM with MCG could be analyzed only on five subjects, however, again, an internal consistency of the physiological entity (HBS) across these modalities indicated the reliability of the proposed method as shown for two subjects in Figures 6 and 7. The beat contours of BSPM showed irregular patches in the time regime of HV interval, possibly due to weak signal strength of HBS reaching the noise floor of the measurement in most of the beat epochs. While the signal treatment and the input SNR of both the modalities were roughly the same, highly resolved and continuously aligned HBS observed in MCG contours could be possibly attributed to the intrinsic characteristic of MCG signals (measured using highly sensitive SQUID sensors) that were relatively less influenced by the conductivity profile of the intervening tissues surrounding the heart (which includes the anatomy of the His bundle as well [Tavarozzi et al., 2002]). The efficiency of this approach was primarily dependent on the prominence of the His bundle spike or bump in the template beat (averaged beat), which was used for fixing the threshold parameters for wavelet thresholding. Several factors affected the notability of His bundle bump such as, the closeness of the bump to the offset of the P wave, level of atrial repolarization, optimal measurement location on the chest and most importantly, the input SNR of the measurement (Sengottuvel et al., 2017; Senthilnathan et al., 2012; Yamada et al., 2003). Our simulation results prescribed an SNR range ~15 dB and an atrial repolarization presented with a magnitude ~1/3rd of the P wave (Type 1 in simulation) to be optimum for recovering the HBS in every beat using this method. This estimation on the adequacy of SNR was consistent with the quantitative assessment performed on the retrieved MCG‐HBS of all the subjects in Table 1. However, this analysis also suggested a scope for further improvement in the input SNR of the signal by pre‐processing MCG data before applying the proposed method. The magnitude limit suggested for PT_a_ was also within the expected range considering independent studies on the measurements of atrial repolarization (Fenici & Brisinda, 2007). The maximum correlation of the extracted HBS that could be achieved using the proposed method was ~70%–80%, even in a high SNR. This indicated a possible distortion in the retrieved signal. Nevertheless, the contribution of these distortions for the purpose of visualizing HBS using contours was insignificant. The conventional use of filters for HBS like that employed in EPS might require extensive evaluation due to the possible imposition of filtering distortions (Agarwal & Gupta, 2013; An & Stylios, 2020) and might mislead the noninvasive identification of HBS. The present work managed the filtering problem by defining the region of interest and appropriately using wavelet thresholding and SSP as demonstrated. Use of projection‐based techniques to selectively remove drift signals whose frequencies overlapped with ramps in the PR segment were reported in literature (An & Stylios, 2020) and it justified the choice of SSP for this purpose of removing atrial components.

It should be admitted that the proposed method has some limitations primarily on its application to cases where significant beat‐by‐beat variations are expected in the HV duration. Second, the method could not be applied on measurement data where the noise floor far exceeds the magnitude of the His bundle signal. The latter situation is a common scenario in most of the experimental conditions irrespective of the fact these measurements are performed inside MSR. Nevertheless, the usefulness of the IDWT‐SSP method and the visualization of HBS in inter‐beat contour as shown in this work could still be acceptable for needy cases where continuous tracking of HBS is expected to be informative like for example, in studying the influence of pharmacological interventions (Su et al., 2020) and follow‐up measurements where a conventional EPS recording involving fluoroscopic radiation may not be advisable (Muresan et al., 2019). There are ample scopes for refinements on the proposed method with respect to the automation of the IDWT process for fixing thresholds settings and adapting the methodology to encounter arrhythmic beats etc.

## 5 CONCLUSION

In summary, a new approach has been presented to track and visualize HBS in every beat through inter‐beat contour maps with sufficient examples taken from MCG of subjects for whom the HV intervals were already known and signal averaging had demonstrated noninvasive determination of HV intervals that were comparable to EPS with an acceptable error limit of agreement. The method applied on BSPM measured on five subjects (of the same MCG group) showed the superiority of MCG in unraveling HBS and were visualized in the inter‐beat contour maps. The utility of this method had also been shown on a healthy subject under moderate exertion. These demonstrations provide confidence in using the technique in various investigations where continuous non‐invasive monitoring of HBS is required.

## AUTHOR CONTRIBUTIONS

Sengottuvel was the originator of the idea and lead investigation on the research problem. Shenbaga Devi provided the conceptual design of the work and guidance. Sasikala reviewed the software codes. Santhosh Satheesh and Raja Selvaraj provided clinical interpretation of the results and critical review of the overall work. All the authors contributed to manuscript compilation. All the authors had read and approved the final manuscript.

## CONFLICT OF INTEREST STATEMENT

There is no conflict of interest for any of the author of this manuscript.

## ETHICS STATEMENT

The research reported in this work was approved by the Institutional Human ethics committee of JIPMER, Pondicherry, JIP/IEC/3/2012/7. All the subjects gave informed consent in participating in the experiment conducting MCG measurements.

## Data Availability

The data used in this analysis is available with the corresponding author and could be obtained upon reasonable request for academic purposes, subject to the approval of the institutions of the authors.
